# Scavenging Rate Ecoassay: A Potential Indicator of Estuary Condition

**DOI:** 10.1371/journal.pone.0127046

**Published:** 2015-05-29

**Authors:** Augustine G. Porter, Peter R. Scanes

**Affiliations:** 1 Coastal and Marine Ecosystems Group, Sydney University, Sydney, NSW, Australia; 2 Estuary and Catchment Science, Office of Environment and Heritage, Sydney, NSW, Australia; Stockholm University, SWEDEN

## Abstract

Monitoring of estuary condition is essential due to the highly productive and often intensely impacted nature of these ecosystems. Assessment of the physico-chemical condition of estuaries is expensive and difficult due to naturally fluctuating water quality and biota. Assessing the vigour of ecosystem processes is an alternative method with potential to overcome much of the variability associated with physico-chemical measures. Indicators of estuary condition should have small spatial and temporal variability, have a predictable response to perturbation and be ecologically relevant. Here, we present tests of the first criterion, the spatio-temporal variability of a potential ecoassay measuring the rate of scavenging in estuaries. We hypothesised that the proposed scavenging ecoassay would not vary significantly among A) sites in an estuary, B) trips separated by weeks, or C) days in a trip. Because not all habitats are present in all estuaries, this test was undertaken in two habitats. When conducted over bare substrate there were occasional significant differences, but no discernible patterns, within levels of the experiment. When conducted over vegetated substrate, days within a trip did not vary significantly, but later trips experienced greater scavenging. This scavenging ecoassay shows potential as a tool for assessing the condition of estuarine ecosystems, and further exploration of this protocol is warranted by implementation in estuaries across a gradient of anthropogenic stress.

## Introduction

Estuaries provide important ecosystem, aesthetic, economic and recreational services, such as coastal protection, nutrient cycling, species diversity, and tourism [1]. They are also critical to the recruitment of many important species [2, 3]. Yet many estuaries are the focus of extensive anthropogenic pressures [4]. Indeed, estuaries are among the most highly eutrofied and impacted environments on Earth [5, 6]. Estuary assessment is therefore of critical importance to demonstrate where management efforts are necessary, to guide such efforts, and to measure their effectiveness where implemented. Furthermore, in cost-constrained situations it is essential for these tools to be cost-effective, quick and reliable [7].

Due to their spatio-temporal variability, estuaries present a unique challenge when attempting to quantify the condition therein. Estuarine organisms live in an intrinsically variable environment, where there are large natural changes in stressors such as temperature, salinity and bed sediments. As a consequence, estuarine organisms must have strategies for coping with stress without adverse effects. These very characteristics, however, mean that it is difficult to see impacts of anthropogenic stress in estuarine environments [8]. Because of the changes that naturally occur in even the least disturbed estuaries [3], multiple indicators are required to accurately quantify their condition [9].

Water quality measures alone have been shown to be inadequate for monitoring the condition of estuaries [10, 11]. Most existing techniques for assessing estuary condition involve intensive collection of chemical parameters or quantifying assemblages of fish and/or invertebrates for indices of biotic integrity, biological traits analysis or other biological indicators, [12–15]. Among the measures of fish and invertebrate assemblages, presence, absence and abundance are commonly used to indicate ecological integrity [16, 17]. However, it has been argued that estuaries, unlike many other ecosystems, do not necessarily rely on high biodiversity to function well, and often support low biodiversity even when in good condition [8].

An alternative strategy is the use of process-oriented measures, or ecoassays [18, 19] to define estuary condition. An ecoassay is a measure of an ecosystem process, which holds predictive power for the broader ecosystem. This focus on the “vigour” of a process increases the temporal relevance of a measure [8]. A good ecoassay is useful in assessing estuaries because, rather than assessing stocks of physical or biological variables, it measures processes important to the functioning of the ecosystem and the organisms living in it. Sampling the processes associated with long-lived organisms has potential to integrate the conditions they have endured in a tractable measure. Fish-based ecoassays have the advantage that fish live in virtually all estuaries, they are relatively easy to identify, they are long-lived and mobile, and they are inherently valuable to the public [10, 20]. Among the potential fish-based ecoassays that could be developed, those focusing on scavenging appear promising. Scavenging; the consumption of carrion before it is made unpalatable by bacteria or microbes, is an ecologically important and widespread phenomenon. While few animals are obligate scavengers, many animals are opportunistic scavengers. Indeed studies have shown that most carnivores and omnivores will partake in scavenging behaviour given the opportunity [21, 22].

Scavenging is a particularly interesting ecosystem process for several reasons. Due to its inherent links with animal assemblages and activity, scavenging is likely to be an excellent indicator of both ecosystem vigour and resilience [23]. In vigorous ecosystems (those with high metabolic activity, productivity and nutrient turnover), the demand for nutrients is likely to be intense across all levels of ecological organisation. There is also strong evidence that scavenging contributes to occasional or opportunistic trophic interactions or “weak links” in a food web which are important for maintaining resilience and stability [24]. Finally, both obligate and opportunistic scavengers comprise a large and diverse portion of omnivores and carnivores in many ecosystems, including estuaries [25]. Scavenging is a pervasive and important ecosystem process performed by a large and diverse group of species, making it an ideal candidate for an ecoassay [21, 23].

The use of bait consumption as a measure of system functioning has some precedents. Britton and Morton [26] asserted; “The present success of marine scavengers can be attributed to human interference in the sea”. VonTorne [27] used a “Bait-lamina test” to assess feeding activity of soil-living animals and Fairweather [18] deployed wood blocks among mangroves and assessed invasion by shipworms (*Teredo* spp.) as an ecoassay. Huijbers et al. [28] deployed fish carcases on beaches to assess the rate of consumption and the assemblage of scavengers as an ecoassay. Webley [29] deployed “carrion platters” in muddy estuaries to assess abundance of and scavenging rates of the Portunid crab *Scylla serrata*. He found that scavenging varied independently of water quality. Scanes, McCartin et al. [30], and NSW Office of Environment and Heritage (author’s unpublished data) used the same techniques described here to compare scavenging rates within and among estuaries. That work found that whilst there was not good discrimination among sites within an estuary, rates of scavenging in estuaries that were independently shown to be in poor condition were much smaller than in those in better condition.

Another advantage of scavenging as an ecoassay is the broad assemblage of species involved. Multi-species metrics/indicators have been shown to better detect a broad range of impacts [14], which is essential in an estuary assessment tool. The process of scavenging in an ecosystem rarely reaches its full potential. The diverse assemblage of opportunistic scavengers [21] allows the potential to remain high, while the actual occurrence remains relatively low [26]. This latent demand for carrion lends itself to an ecoassay as the potential vigour of the process can be measured on-demand. Additionally, live prey need not be mimicked, and long exposure times (at least 24 hours) are not problematic [31].

If a measure is to be useful as an ecoassay it must be robust to spatio-temporal variability (when ecosystem condition and water body are held constant) and it must correlate with an important aspect of ecosystem condition or health. In this study we examine the first pre-requisite, and also determine the most appropriate sampling strategy by measuring scavenging rates over various spatial and temporal scales. We tested the hypothesis that scavenging rates would not vary significantly among A) sites within an estuary, B) trips within a season and C) days within a trip. We did not test any correlation between scavenging rate and estuary condition. That important step will need to be undertaken in a separate study.

## Methods

### Ethics Statement

No animals were removed from their original habitat, harmed or retained during this study. Only videos and records of bait consumption were taken. Animal ethics approval was provided according to State Legislation. Field permit for research was issued by New South Wales Department of Primary Industries, Fisheries Office.

### Bait Deployment

Baits were Individually Quick Frozen (IQF) pilchards (*Sardinops sagax*), purchased from a commercial supplier. These baits were chosen as a standard attractant of consistent quality which could be easily exposed to scavengers, and subsequently retrieved without disintegrating. Pilchards are a commonly used bait known to attract a wide variety of species [32].

All work was done in Lake Macquarie NSW, Australia (33.09° S, 151.61° E) which is a wave dominated barrier estuary [33] that experiences micro-tidal fluctuations (approx. 5–10 cm tides) due to its permanently open, but relatively restricted, channel to the sea. It is the largest coastal lagoon in New South Wales, with a surface area of approximately 115 km^2^ [33]. The lake supports extensive beds of seagrass, most prominently *Zostera muelleri*, *Posidonia australis*, *Ruppia maritima* and *Halophila ovalis*, [34] which occupy an area of 13.4 km^2^ [33]. Lake Macquarie is generally considered to be in good condition [11]. It represents a suitable site to provide unconfounded tests for the effects of non-anthropogenic factors (site, season and day) on rates of scavenging.

**Fig 1:**
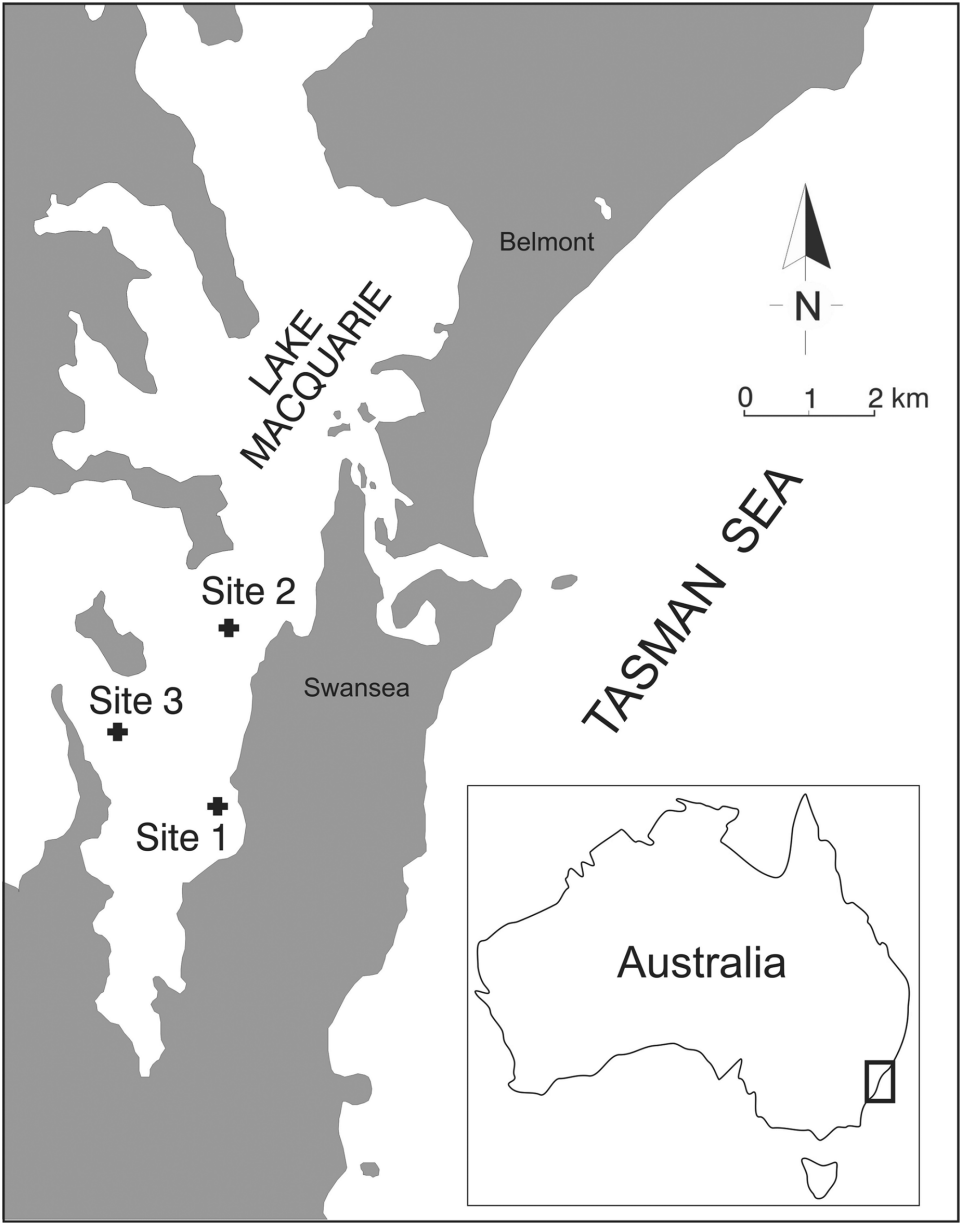
Study sites within Lake Macquarie, and location on the coastline of NSW, Australia.

The experimental design was to present baits to scavengers in each of two habitats (vegetated and bare substrata) at each of three sites within Lake Macquarie and measure the amount of bait consumed in a fixed time period. The three sites (1: Cams Wharf, 2: Swansea and 3: Wolstoncroft, see Fig 1) were similar in depth, exposure and substrate. At each site an area of vegetated substrate (primarily *Z*. *muelleri*, interspersed with *H*. *ovalis* and sparse *P*. *australis*) and an area of bare substrate were identified at a depth of 1.5–3 m, but separated from each other by at least 50 m.

Eight baits were presented within each habitat at every site on each day of sampling. During the study there were 3 sampling occasions (trips) and within each trip, baits were presented on 3 consecutive days. The order (and therefore time of day) that the sites were visited was randomised to avoid time-of-day biases. All sampling was done between 26 October and 16 November 2010 (austral spring).

Baits were presented on scavenging trays (0.5 m diameter) consisting of a fine-mesh base with weighted wire frame and monofilament line to a surface float with a unique identifier. The mesh was designed to rest on the substrate and suppress vegetation around the bait, ensuring they were not hidden from potential scavengers, and to retain pieces of bait that were removed but not consumed. The baits were pre-weighed, thawed, pilchards secured to the tray using a plastic cable-tie through the eye socket and a second cable-tie around the body.

The 8 trays were deployed at least 10m apart within the same contiguous habitat type. On the first day of sampling, trays were left in place for 30 minutes. After review of the results, it was decided that to increase consumption and reduce variability among replicates, the soak time needed to be longer and all subsequent deployments were between 60 and 70 minutes. After retrieval, any remaining bait was removed from the trays and weighed (0.01 g). All missing bait was presumed to have been consumed.

A random subset of trays were attached to underwater video cameras to record the species that were consuming baits. 15 randomly chosen deployments in each habitat were recorded. Cameras (Canon HG21 f1.18 4.8–57.6 mm zoom lens set at 4.8 mm with a 0.5 X HD wide angle attachment) in cylindrical sealed housings were attached to arms that held the tray 600 mm from the camera. The field of view encompassed an area of approximately 0.6 m to either side and above the tray, and 2–3 metres behind the tray (depending on water clarity). Data collected from the underwater videos were analysed for species observed (to lowest taxonomic level possible), time to first feeding on the bait, and time from first feeding until bait totally consumed.

### Data Analysis

The amount consumed was calculated as the difference in weight from before to after deployment and standardised by dividing by starting weight. This was expressed as the proportion consumed. Consumption rates were calculated by dividing the proportion consumed by the time of deployment (min). Two instances of minor negative consumption due to measurement error were corrected to 0.

Data from bare and vegetated substrata were analysed separately. All data were analysed using analysis of variance to test for differences between sites, trips and days using the statistical program GMAV [35]. SNK post-hoc tests were used to identify differences among means where ANOVA indicated interactions among factors. Prior to analysis, data were tested to ensure that they met the assumptions required for ANOVA [36] and, if necessary, were transformed. Data were not tested to satisfy normality assumptions because the sample size was large and ANOVA is quite robust to non-normal data [36].

To test if changes in deployment time after the first day (day A) had an effect on measured scavenging rates, day A was compared to days 2 and 3 of trip 1 (days B and C) using t-Tests with Welch’s correction to degrees of freedom where variances were unequal.

To determine the most appropriate sampling duration, the data were initially analysed for variations among site, trip and day. Site was a random factor with 3 levels. Trip was a random factor with 3 levels. Day was a random factor with 3 levels, nested in trip. The replicates (n = 8) were individual pilchards exposed to scavenging. In this model, there is no appropriate mean square denominator to test for the effects of trip without pooling interaction factors. Prior to analysis, data were tested for homogeneity of variance using Cochran’s test. All data required ArcSin transformation. Even after transformation, Cochran’s test for homogeneity of variances returned a significant result for data collected at bare substrate (C = 0.1500, P < 0.01). We acknowledged this and continued with the analyses because the sample size was large (N>60) and Sokal and Rohlf [37] and Underwood [36] suggest that in these cases, deviation from the mean is likely to be small and significance of results is not compromised.

## Results

**Fig 2:**
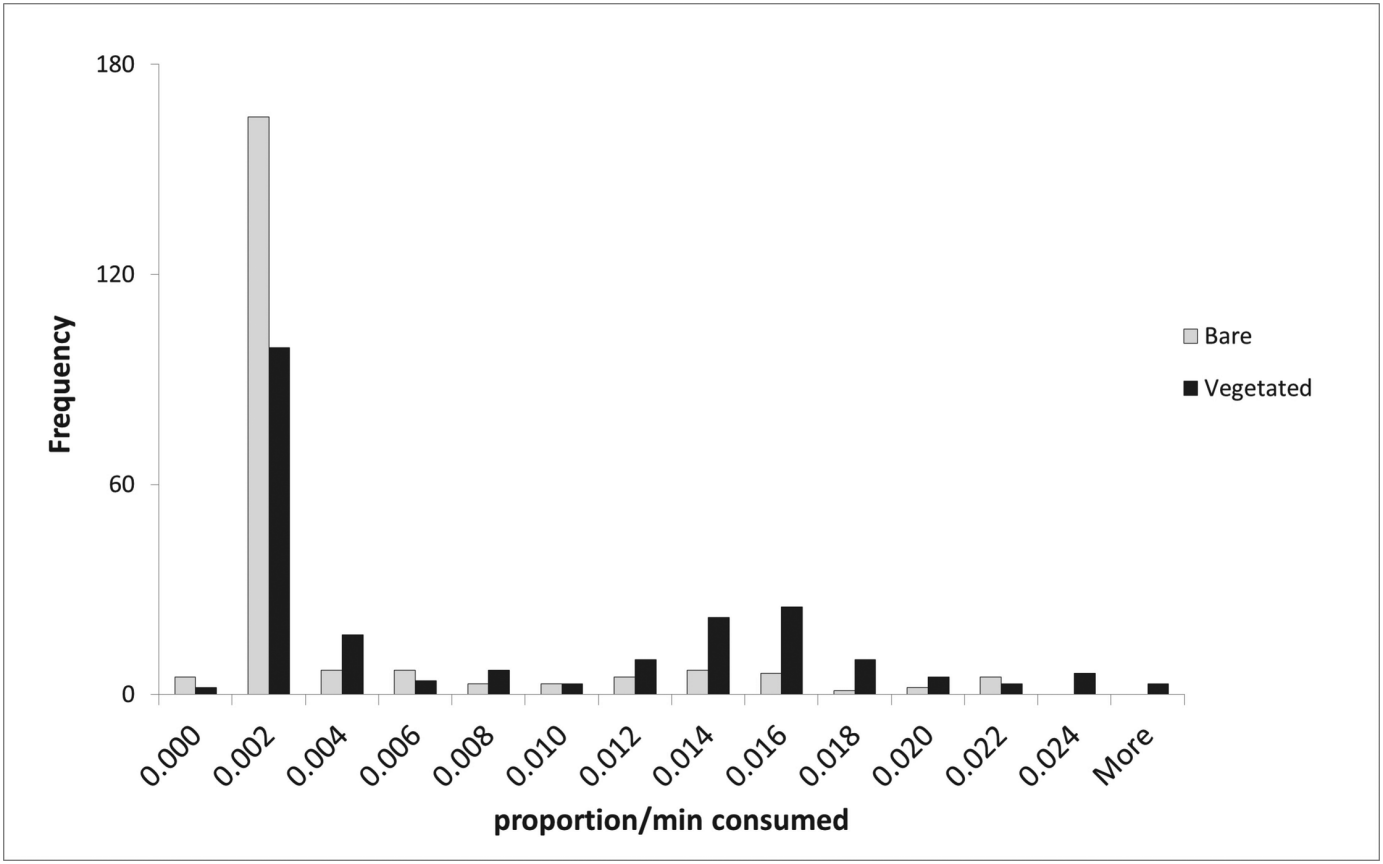
The majority of scavenging data are at or near zero resulting in a skewed distribution.

The data were highly skewed, with the majority of data at or near to zero (Fig 2). The high frequency of results in the 0.002 range is likely due to sampling procedures such as the loss of eyes from baits because of the method used to secure baits to the scavenging tray.

Day A (30 min soak) was not statistically different from day B or C (60 min soak) at any site (Table 1). Therefore, it is assumed that the adjustment in protocol after day A does not significantly affect the results. The authors acknowledge that repeated tests on the same dataset greatly increase the type 1 error rate [36]. This is normally addressed by adjusting α with a Bonferroni-type correction [37]. However, these corrections increase the Type 2 error rate (if the null hypothesis is rejected). In this instance we have retained the null hypothesis (no possibility of Type 1 error) despite the elevated probability of Type 1 error. Furthermore, because α remains at 0.05, there is no elevated Type 2 error rate. The authors are confident that this result, in addition to the fact that all results are expressed as rate of consumption, is sufficiently conservative that the results may be included in further analyses.

**Table 1:** Results of t-Test on scavenging rates on day A vs days B & C at each site x habitat combination.

Site	Habitat	DF	t-statistic	Significance	P
**1**	bare	8	0.28	Not Significant	0.79
**2**	bare	22	-0.79	Not Significant	0.44
**3**	bare	8	1.28	Not Significant	0.57
**1**	veg	22	-0.44	Not Significant	0.66
**2**	veg	7	1.37	Not Significant	0.21
**3**	veg	20	-1.02	Not Significant	0.32

Degrees of freedom adjusted as per t-test assuming unequal variances (Welch Approximate Degrees of Freedom test).

All levels of the experiment were random in order to ensure the generality of the null hypothesis that scavenging rate would vary among sites, trips and days. Despite the levels being random it is still useful to examine the nature of the post-hoc SNK tests for any consistent pattern in direction of differences. If the differences are not consistent in direction, we may conclude that they were probably caused by stochastic events that cannot be determined with the current data. However, if they are consistent in direction, it would indicate an underlying pattern which may warrant further examination.

### Bare Habitat

**Fig 3:**
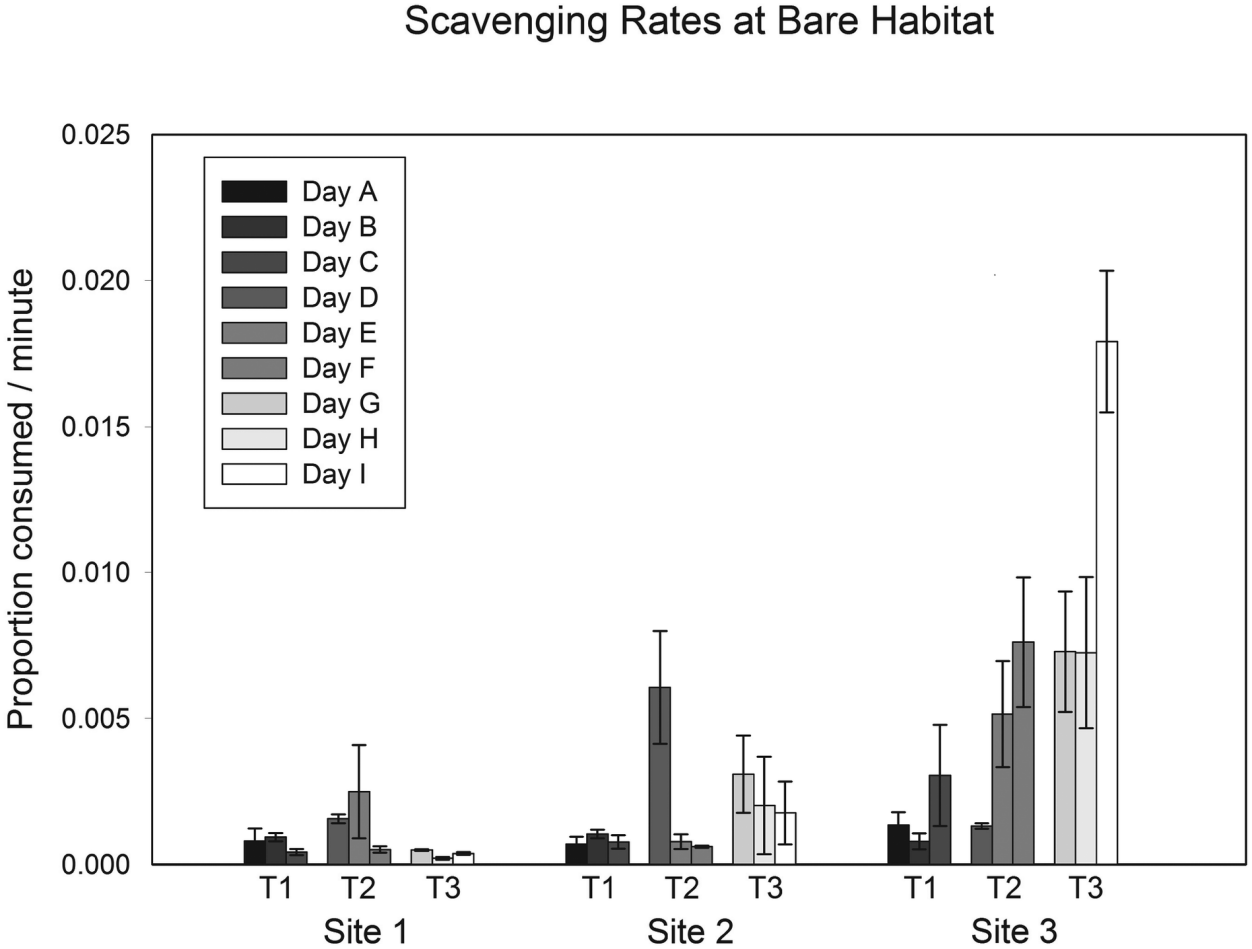
Scavenging rate at bare habitat (mean ± 1 SE). Differences between Trips and Sites could not be generalised due to interactions between these two levels.

Analysis of scavenging data at bare habitat indicate that there is significant variability in scavenging rates among days within trips, but the differences were not consistent among trips or sites (siXda(tr) interaction, Table 2). Using the Cornfield Tukey rules the design does not yield a denominator term for the factor “trip” because pooling of lower terms was not possible with the data collected at bare habitat. Post-hoc SNK tests revealed that there were significant differences among the days of a trip on only two of the nine trip x site combinations, (Fig 3). There were also two instances of significant site x trip interaction. This low frequency of occurrence would suggest infrequent stochastic events rather than consistent differences.

**Table 2:** Analysis of variance on scavenging rate over Bare habitat expressed as proportion of bait consumed per minute.

Source	SS	DF	MS	F	P
**si**	169.11	2	84.56	3.93	0.11
**tr**	52.64	2	26.32	0.00	NO TEST
**da(tr)**	25.79	6	4.30	0.47	0.81
**siXtr**	86.02	4	21.51	2.73	0.11
**siXda(tr)**	108.89	12	9.06	3.97	<0.001
**Residual**	431.89	189	2.29		
**Post-Hoc Tests (= indicates no significant difference)**					
si 2 tr 2 (da D > E** = F)					
si 3 tr 3 (da G = H < I**)					
si 3 (tr 1 = 2 < 3*)					
tr 3 (si 1 = 2 < 3**)					

si = “site” tr = “trip”, da = “day”. N = 216. Only significant (*, P < 0.05) and highly significant (**, P < 0.001) Post-Hoc test results are shown.

### Vegetated Habitat

**Fig 4:**
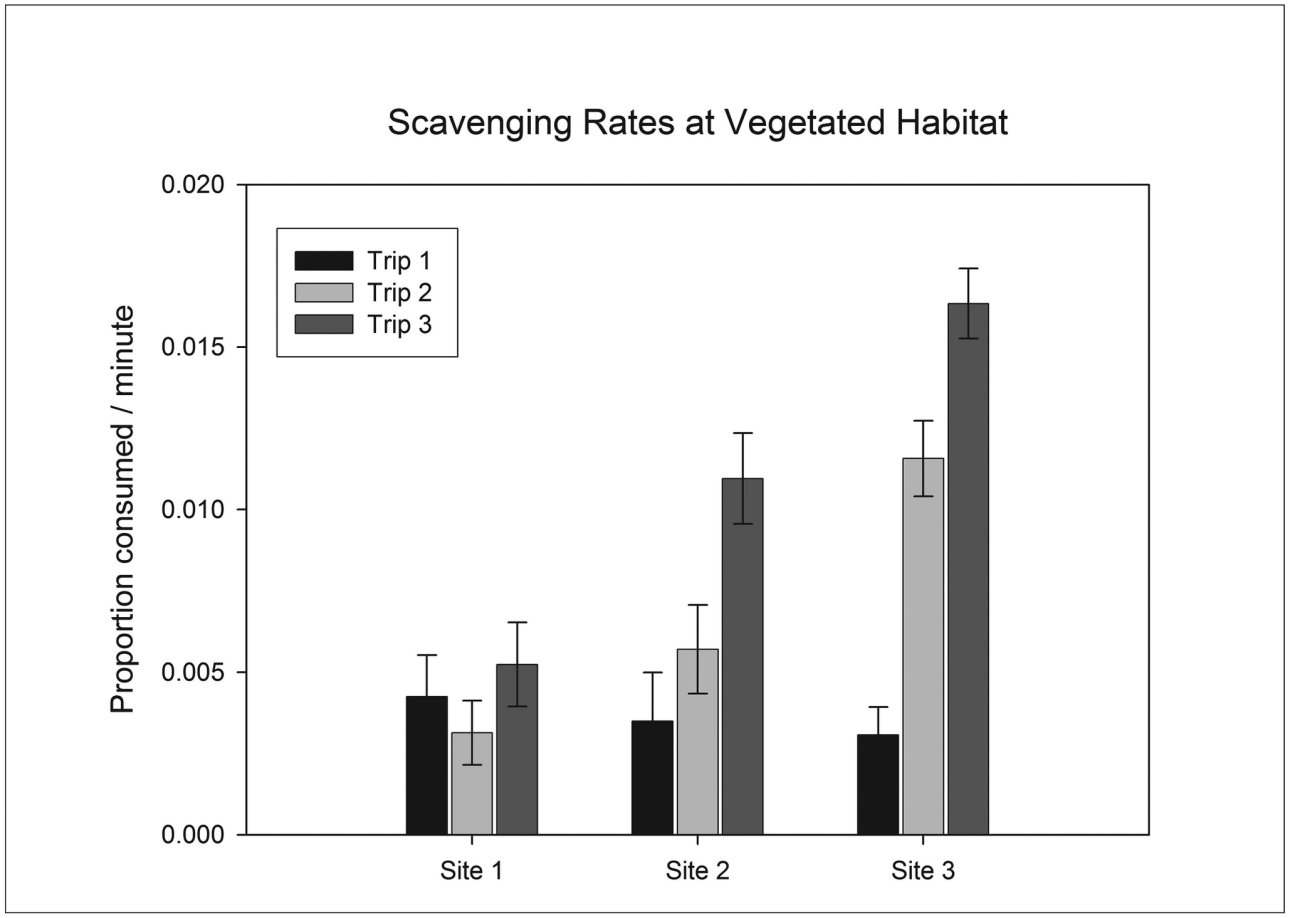
Scavenging rate on vegetated habitat (mean ± 1 SE). Days did not differ significantly within trips at any site and are not differentiated in this figure.

Analysis of data collected over vegetated habitat indicated that days did not differ significantly within any trip. There was, however, an interaction between sites and trips (Table 3). SNK tests revealed that at one of the three sites trips did not differ, while at the other two sites later trips had significantly higher rates of scavenging (Fig 4). Additionally, on one of the three trips sites did not differ, while on the other two there were significant differences among sites.

**Table 3:** Analysis of variance on scavenging rate over Vegetated habitat expressed as proportion/min. si = “site” tr = “trip”, da = “day”.

Source	SS	DF	MS	F	P
**si**	179.54	2	98.73	3.05	0.16
**tr**	252.8	2	126.14	0.00	NO TEST
**da(tr)**	71.02	6	11.84	1.97	0.15
**siXtr**	129.39	4	32.35	5.39	0.01
**siXda(tr)**	72.08	12	6.01	1.28	0.23
**Residual**	886.81	189	4.69		
**Post-Hoc Tests (= indicates no significant difference)**					
si 2 (tr 1 = 2 < 3*)					
si 3 (tr 1 < 2** = 3)					
tr 2 (si 1 = 2 < 3**)					
tr 3 (si 1 < 2* < 3**)					

N = 216. Only significant (*, P < 0.05) and highly significant (**, P < 0.001) Post-Hoc test results are shown.

### Video Analysis

Video recordings of a subset of the scavenging trays captured a total of 11 species feeding at the baits, 9 fish, 1 crab and 1 sea star (Table 4). Seven species were filmed scavenging in bare habitat and 8 in vegetated (Tables 4 and 5), but only 4 were recorded at both habitats. More baits were visited in vegetated habitats (Table 5) and the number of species per videoed bait was greater in vegetated habitats (2.7 spp/bait) than bare (1.7 spp/bait). Scavenging in vegetated habitats was dominated by eastern striped trumpeter (*Pelates sexlineatus*) and tarwhine (*Rhabdosargus sarba*), with each present at 9 of the 10 deployments visited by scavengers (Table 4). *R*. *sarba* was present as often as *P*. *sexlineatus*, but the latter appeared to consume far more bait. At bare habitat *P*. *sexlineatus* and *R*. *sarba* were much less prevalent (present at 2 and 1, respectively, of the 6 deployments visited by scavengers) and the yellow fin bream (*Acanthopagrus australis*), made up a greater proportion of the assemblage attending baits (4 of the 6).

**Table 4:** Species visitation frequency.

Species	Bare	Vegetated
**Fishes**		
***Pelates sexilineatus***	0.33	0.90
***Acanthopagrus australis***	0.67	0.30
***Rhabdosargus sarba***	0.17	0.90
***Pagrus auratus***	0	0.20
***Monocanthus chinensis***	0	0.10
***Silago ciliata***	0.17	0
***Urolophus spp***	0.17	0
***Tylosurus gavialoides***	0.17	0.10
***Pseudorhombus spp*.**	0	0.10
**Seastar**		
***Coscinasterias muricata***	0.2	0
**Crab**		
***Portunus pelagicus***	0	0.20

For the videoed baits (N = 15 for each habitat) that were visited by fish, proportion of times that each species was observed in each habitat.

**Table 5:** Proportion of baits where fish were observed, total number of species observed, and, for the baits visited by fish, average number of species per bait.

	Bare	Vegetated
**Proportion with fish**	0.43	0.67
**# of Species visiting baits**	7	7
**Average # of Spp. (excl. un-visited baits)**	71.02	6

Baits in bare habitats were visited by a large range of fish but relatively little feeding occurred. The time to first consumption of bait was much greater in bare habitats (consumption only occurred in 1 out of 6 visited baits, after 30 min) than vegetated (average time to first consumption 8 min).

## Discussion

We hypothesized that scavenging rates would not vary significantly across A) sites within an estuary, B) trips within a season and C) days within a trip. We found there to be occasional significant variation at the site and trip level at both bare and vegetated habitats. Furthermore, we demonstrated that at vegetated habitats, days within a trip did not vary, indicating that sampling on consecutive days is not necessary at this habitat.

The two instances of significant variation among days within a trip at bare habitat were in different directions. In one, scavenging decreased on later days, while it increased on later days in the other. These differences suggest that there is not an underlying trend in differences among days, but stochastic differences that we cannot distinguish with our current dataset. Similarly, the interactions between trip and site are too few to discern any underlying pattern.

The two instances of significant differences among trips at vegetated habitat were in the same direction, with later trips having greater scavenging. This result suggests that there may be an underlying factor influencing scavenging rates across trips. However, as our design tested trip as a random factor we cannot interpret causes of these differences. The two instances of significant differences among sites at vegetated habitat were in the same direction with site 3 consistently having the greatest scavenging rate. While these differences in scavenging rate are significant in the context of the current experiment in one estuary, it is quite possible that in the context of estuaries of different condition they would be insignificant.

Video recordings of a subset of the scavenging trays indicate that while a wide variety of fishes and invertebrates visit baits deployed over vegetation, the eastern striped trumpeter (*P*. *sexlineatus*) and tarwhine (*R*. *sarba*) are the most common, and dominate the assemblage in terms of abundance (see S1 Table). Observation of the videos indicates that while *R*. *sarba* is present as often as *P*. *sexlineatus*, the latter consume far more bait. At bare habitat, the trays seemed to attract a few individuals of a wide variety of species, but little feeding occurred. At bare habitat, *P*. *sexlineatus* are much less prevalent (than at vegetated) and yellow fin bream (*A*. *australis*) make up a greater proportion of the assemblage attending baits. Only one of the videoed baits at bare habitat experienced significant scavenging (by *A*. *australis* and *P*. *sexlineatus*), so it is not possible to conclude which species are responsible for the majority of consumption at this habitat.

It is possible that the scavenging rate measured in these trials could simply reflect the assemblage of fishes and other organisms present at a time and place. This would defeat the purpose of measuring scavenging rate, as opposed to simply measuring fish assemblages. However, several of the video recordings show *P*. *sexlineatus* near baits but not feeding. Optimal foraging theory suggests that in systems where food is less available, individuals will spend more time feeding on available prey items [38, 39]. Additionally, *P*. *sexlineatus* has been shown to exhibit greater boldness when food is scarce [40]. These findings reinforce the likelihood that the current ecoassay is not simply a measure of fish abundance, but instead is a result of scavenger behaviour, underpinned by elements of ecosystem condition.

Differences in scavenging rates among habitats are presumably due to scavenger habitat preferences rather than condition, as the two habitats are adjacent to each other. *P*. *sexlineatus*, for example, has a very strong preference for vegetated habitats [30]. But these behavioural factors remain interesting as a potential explanation of spatio-temporal changes within a habitat.

In this context the measure of scavenging rate over bare habitat is not necessarily inferior to scavenging rate over vegetated habitat as a potential ecoassay. Indeed, scavenging rate at bare habitat could potentially be more responsive to ecosystem changes, as scavenging appears to be distributed more evenly across the assemblage of fishes and invertebrates present. However, if rate of consumption at bare habitat is to be used, it is clear that a larger number of samples (probably from multiple days) will need to be collected to counteract the greater variability in scavenging rates at that habitat type.

Careful consideration should be given to choice of habitats if this protocol is further explored as a potential indicator of estuary condition. The results of ecoassays of vegetated habitat could be confounded if the length and/or density of leaves physically blocks fish from approaching the bait. Despite the scavenging trays used in this study effectively suppressing the surrounding aquatic vegetation in the immediate vicinity, they may not effectively present the bait for scavenging in some deep beds of *Z*. *muelleri*, *P*. *australis* or other long vegetation.

There is potential that repeating this protocol over consecutive days would result in either satiation of scavengers in an area, or learned feeding responses, either of which could bias the results. The scarce evidence available suggests that satiation is not likely to be an issue. For Sticklebacks (*Spinachia spinachia)* which are similar in size to *P*. *sexlineatus*, 24 hours was enough for fish to resume feeding after satiation [41]. Additionally, the data show no significant effect of day on the rate of consumption at vegetated habitat. The two instances of days within a trip differing significantly at bare habitat did so in opposite directions (Table 2). Therefore, satiation or learned responses are not taken to be an important factor in driving scavenging rate between days. It is assumed that fishes and other scavengers do not have the memory or ability to quickly learn any patterns of deployment greater than a day.

Seasonality could have contributed to the trend of greater scavenging rates on later trips as water temperature increased. Seasonality is an interesting and important possibility that will need to be investigated if this procedure is to be linked to estuary condition as an ecoassay. In this instance, however, the entire campaign of 3 trips was completed over the period of 4 weeks in the austral spring. This would indicate that seasonality was probably not a major factor.

Rain records suggest a trend of greater scavenging on trips which were less rain affected. In the 24 hours prior to Trip 1, 2 and 3, rainfall totals were 61.4 mm, 48.4 mm and 40 mm respectively (Bureau of Meteorology, Swansea station ID 061377). Turbidity has been shown to significantly affect the success rate of piscivorous fish [42]. It is feasible that turbidity resulting from catchment inputs could affect the ability of estuarine generalists such as *P*. *sexlineatus* and *A*. *australis* to locate deployed baits and begin feeding. This in turn could affect rates of scavenging and should be controlled for in future studies.

The variability and zero-inflated nature of the data was not anticipated. Scavengers tended to focus on baits that had already been fed on, ignoring intact replicates. This behaviour resulted in almost binary results (baits either entirely consumed or not touched), contributing to high variability and the inability to pool data. We were not able to reject our null hypothesis, except at the day level over vegetated substrate. However, there are several useful conclusions to be drawn from the experiment. Firstly, the variability among sites within an estuary, trips in a season and days in a trip is now quantified. Secondly, we have shown that replication at the day level over vegetated substrate is not necessary. Thirdly, we have quantified the rate of scavenging in Lake Macquarie in spring for future comparison with estuaries of different condition.

While there were occasional significant differences among the levels of this experiment, it is entirely possible that these differences would be insignificant in the context of among-estuary differences. Indeed, using the same methodology, Scanes et al [30] demonstrated that scavenging rates among estuaries in the Port Stephens, NSW region differed significantly.

Our study does not provide any direct assessment of whether scavenging rates differ in a predictable pattern among estuaries subject to human disturbance. To help formulate hypotheses about scavenging rates, we provide a conceptual framework of how scavenging rates might change under various impacts (Table 6 for summary).

**Table 6:** How might scavenging rate change with different impacts? “Indicator scavenging rate” refers to the rate of consumption of experimentally deployed carrion.

Disturbance Form	Scavenger Abundance	Scavenging Rate
Toxic Stress	**↑**	**↓**
Removal of Piscivores	**↑**	**↑**
Removal of Invertivores	**↓**	**↓**
Eutrophication	**↑**	**↓**
Loss of seagrass	**↓**	**↓**

In assessing how scavenging might be used as an indicator we have considered the ecological processes that might be disturbed by different impacts and the consequent changes to scavenging rates. These predictions are based on a number of assumptions that have emerged from the work so far:

- The primary consumers of introduced carrion that we observed were middle trophic level fish.
- These fish are rarely piscivores and are more likely to rely on benthic macroinvertebrates as primary prey [43]. They are therefore facultative scavengers and consumption of carrion is influenced by overall need for food.
- There is experimental evidence for one of the main species involved (the eastern striped trumpeter *Pelates sexlineatus*) indicating that boldness increases as food becomes scarce [40]. Consumption rates will therefore be influenced by competition for food influencing satiation and boldness.
- “Increased” and “decreased” consumption rates are in comparison to undisturbed control locations.
- “Indicator scavenging rate” herein refers to the rate of consumption of experimentally deployed carrion, not the overall process of scavenging in the ecosystem.

### Model 1: Toxic stressors

Disturbances which increase disease and death of fish from non-predator related factors (e.g. climate change, toxins [25]) increase biomass of natural carrion available to consumers. This encourages scavenging but means that food is more plentiful. Hypothesis: Scavengers should be more abundant, but indicator scavenging rates decreased.

### Model 2: Removal of higher trophic levels (piscivores)

According to trophic cascade theory, this should lead to an increase in abundance of middle trophic levels and a consequent increase in competition for food. Hypothesis: Scavengers should be more abundant, and indicator scavenging rates increased.

### Model 3: Removal of middle trophic levels (invertivores)

This should lead to a relative increase in invertebrate food resources and reduced completion for food among the remaining consumers. Hypothesis: Scavengers should be less abundant and indicator scavenging rates decreased.

### Model 4: Eutrophication

Eutrophication should lead to an increase in algal growth, with consequent increase in plant detritus. Many invertebrates can utilise algae and detritus as food sources and so should become more abundant, reducing competition for food among middle trophic level fish. Hypothesis: Scavengers should be more abundant, but indicator scavenging rates decreased.

### Model 5: Loss of seagrass

The most vigorous scavengers were eastern striped trumpeter which are strongly associated with seagrass habitats [30]. Loss of seagrass would significantly reduce their abundance. Hypothesis: Scavengers should be less abundant, and indicator scavenging rates decreased.

Scavenging rate shows promise as a tool in estuary assessment. However, several tests are still required to prove, or disprove, its usefulness. The tool must be trialled in estuaries across a gradient of conditions, and in different seasons. These steps should examine the consistency and generality of scavenging rate, giving us a better understanding of its potential applications.

## Supporting Information

S1 Table Video Summary. (XLS)

S2 Table Dataset. (XLSX)
